# ﻿Three novel Ascomycota (Saccharomycetes, Saccharomycetales) yeast species derived from the traditional Mexican alcoholic beverage Pulque

**DOI:** 10.3897/mycokeys.109.123870

**Published:** 2024-10-09

**Authors:** Chunyue Chai, Dan Lu, Jinli Liu, Eentao Wang, Xuemei Han, Fengli Hui

**Affiliations:** 1 School of Life Science, Nanyang Normal University, Nanyang 473061, China Nanyang Normal University Nanyang China; 2 Research Center of Henan Provincial Agricultural Biomass Resource Engineering and Technology, Nanyang 473061, China Research Center of Henan Provincial Agricultural Biomass Resource Engineering and Technology Nanyang China; 3 Departamento de Microbiología, Escuela Nacional de Ciencias Biológicas, Instituto Politécnico Nacional, Mexico City, Mexico Escuela Nacional de Ciencias Biológicas, Instituto Politécnico Nacional Mexico City Mexico; 4 Ministry of Education Key Laboratory for Ecology of Tropical Islands, Key Laboratory of Tropical Animal and Plant Ecology of Hainan Province, College of Life Sciences, Hainan Normal University, Haikou 571158, China Hainan Normal University Haikou China

**Keywords:** Morphology, phylogenetic analysis, Pulque, taxonomy, yeast

## Abstract

The abundant variety of yeasts and their diverse applications have essential roles in traditional Mexican alcoholic beverage fermentation processes. During our investigation of yeast diversity associated with Pulque, 41 yeast strains were characterized. Among them, 31 strains were eight known species belonging to seven genera. According to morphological and phylogenetic analyses (ITS and LSU rDNA), ten unidentified yeast strains were identified to be three novel species and proposed: *Starmerellaelongatum***sp. nov.**, *Kazachstaniaparagamospora***sp. nov.** and *Pichiateotihuacanensis***sp. nov.** Our study has resulted in the isolation of yeast species not previously detected in Pulque, expanding our knowledge of the diversity of its associated yeast communities.

## ﻿Introduction

Alcoholic beverages arose across various geographical regions over the course of human civilization (McGovern et al. 2004; Mishra et al. 2021), and some traditional fermentation procedures are still maintained in specific regions, like sweet fermented rice (mijiu or laozao) in China (Tian et al. 2022) and Pulque in Mexico (Escalante et al. 2016). Previously, several investigations were conducted into alcoholic beverages (McGovern et al. 2004), their medical functions (Dutfield 2010; Egea et al. 2015), their relationships with tradition and ethnic habits (Mishra et al. 2021) and local culture (Egea et al. 2016), fermentation substrates and protocols (McGovern et al. 2004). However, studies on the microbiome in the traditional alcohol beverages are still insufficient (Maicas 2020; Motlhanka et al. 2020), considering their high diversity and wide geographic distribution.

Pulque is a traditional Mexican alcoholic beverage produced by fermenting fresh sap collected from the hearts of mature, uncooked *agave* plants, including *Agaveamericana*, *A.atrovirens*, *A.ferox*, *A.mapisaga*, and *A.salmiana* (Sánchez-Marroquín et al. 1967). This beverage is produced, sold, and consumed in popular districts of Mexico City and surrounding rural areas. To produce this drink, freshly harvested agave nectar is transported in wood barrels or bags made of goat skins and then transferred into larger barrels for fermentation. The Pulque fermentation process is batch non-stirred and conducted under non-aseptic conditions, varying in duration from a few hours to overnight. The microorganisms that occur naturally during sap accumulation in the *cajete* cavity of maguey (agave) plants, along with those acquired during collection, transport, seed preparation, and manipulation (Lappe-Oliveras et al. 2008; Escalante et al. 2016), also involved in the fermentation process, are diverse and complex.

Studies delving into Pulque-associated microbiota identified an array of associated bacterial and yeast species. These microorganisms generate three unique metabolic products during Pulque fermentation: lactic acid, ethanol, and extracellular polysaccharides (EPS). Regarding the yeast diversity in Pulque, *Saccharomyces* and non-*Saccharomyces* species have both been found and proposed as necessary for the production of ethanol, amino acids, vitamins, and volatile flavor compounds contributing to the sensory properties of the beverage (Lappe-Oliveras et al. 2008). Given that the fermentation of Pulque relies on the natural microbiota, different yeast strains may be present in Pulque products from different sites. Therefore, the diversity of associated yeasts is worth investigating.

To investigate the diversity of yeast in Pulque, we collected six Pulque samples in the autumn of 2015 from Mexico, from which different yeasts were isolated. Based on DNA sequence comparisons and phenetic characteristics, the majority of isolates were identified as characterized species. Among them, ten unidentified yeast strains were identified. To examine their taxonomy further, phylogenetic analyses based on integrated the internal transcribed spacer (ITS) region and the D1/D2 domain of the large subunit (LSU) rRNA gene sequences were performed. Both morphological characteristics and molecular evidence suggest that these yeasts represent three separate species of *Kazachstania*, *Pichia*, and *Starmerella* genera, which are described in detail in this study.

## ﻿Materials and methods

### ﻿Yeast isolation

Six homogenized Pulque samples were obtained from two “*pulquerías*” (Pulque stores) in 2015 in two different Mexico regions: Teotihuacan in the State of Mexico (19°41'N, 98°50'W) with an altitude of 2377 m, and Mexico City in the State of Distrito Federal (19°28'N, 99°09'W) with an altitude of 2400. Pulque samples were fermented for approximately 36 to 48 hours. We removed approximately 10 mL of each Pulque sample into different sterile plastic bags using long-handled spoons. Samples were then transported on ice to the laboratory. Yeasts were isolated from Pulque samples using yeast extract-malt agar (YMA, composed of 1% glucose, 0.3% yeast extract, 0.3% malt extract, 0.5% peptone, and 2% agar, w/v) supplemented with 0.02% chloramphenicol. Each Pulque sample was serially diluted into a sterilized saline solution (0.9% w/v; NaCl). Subsamples of 100 μL were spread on YM plates and incubated at 25 °C for 3~7 days. The yeast colonies were purified through repeated streaking on YM plates followed by incubations at 25 °C. The isolated strains were maintained at 4 °C for short-term preservation and at −80 °C as 20% glycerol (w/v) stocks for long-term preservation.

### ﻿Morphological and physiological characteristics

Morphological observations and metabolic examinations adhering to the standard yeast description profile were performed as previously reported (Kurtzman et al. 2011). Assimilation tests for carbon and nitrogen sources were conducted in liquid media. Strains were starved before nitrogen assimilation tests. All assimilation tests were performed in duplicate, and the results were recorded following 5 and 21 days of incubation. Strains were examined for ascosporulation on agar media prepared as follows: YM, 1% malt extract, 5% malt extract, corn meal, V8 (1: 9) agar, and yeast carbon base supplemented with 0.01% ammonium sulfate (YCBAS; 1.1% yeast carbon base, 0.01% ammonium sulfate, and 1.8% agar), individually or by combining strains pairwise and incubating at 17 °C and 25 °C for up to 4 weeks.

### ﻿Molecular phylogenetic analysis

Genomic DNA was extracted using an Ezup Column Yeast Genomic DNA Purification Kit according to the manufacturer’s instructions. The ITS region and the D1/D2 domain of the LSU rRNA gene were amplified via PCR and sequenced using the ITS1 and ITS4 (White et al. 1990) or NL1 and NL4 (Kurtzman and Robnett 1998) primer pairs, respectively. Each 25 µL PCR mixture included 21 µL PCR-grade water, 1 µL DNA template, 1.5 µM of each primer and 1 µL PCR Master Mix (2×; 0.05 u µl^-1^ of *Taq* DNA polymerase, reaction buffer, 4 mM MgCl_2_, 0.4 mM of each dNTP; Sangon Biotech). PCR reactions were carried out according to the following conditions: an initial denaturation step of 2 min at 95 °C, followed by 35 cycles of 30 s at 95 °C, 55 °C for 30 s, 72 °C for 40 s, with a final extension of 10 min at 72 °C (Lv et al. 2020). PCR products were directly purified and sequenced by Sangon Biotech Inc. (Shanghai, China). We determined the identity and accuracy of the newly obtained sequences by comparing them to sequences in GenBank and assembled them using BioEdit (Hall 1999). Newly obtained sequences were submitted to GenBank (https://www.ncbi.nlm.nih.gov/genbank/).

Phylogenetic relationships between the novel species and their close relatives were characterized using a combined ITS and LSU sequence dataset. *Saccharomycescerevisiae* CBS 1171^T^, *Wickerhamiellasorbophila* NRRL Y-7921^T^ and *Schizosaccharomycespombe* CBS 356^T^ were utilized as respective outgroups. Except for the sequences acquired in this study, the reference sequences were downloaded from GenBank (Suppl. materials S1–S3). Sequences of the individual loci were initially aligned using MAFFT v 7.273 (https://mafft.cbrc.jp/alignment/server/large.html) (Katoh et al. 2019) with the G-INS-I algorithm. Aligned sequences of the different loci were concatenated using PhyloSuite v. 1.2.2 (Zhang et al. 2020). Alignments were enhanced through manual gap adjustments. Ambiguously aligned regions were disqualified prior to analysis. Phylogenetic trees were constructed using the maximum likelihood method in MEGA 7.0 (Kumar et al. 2016). The evolutionary distance was determined using the Tamura-Nei model with a gamma rate distribution and invariant sites. MrModeltest 2.3 software was utilized to determine the best-fit evolution model of the dataset for Bayesian Inference (BI), which was constructed using MrBayes 3.2.5 (Ronquist et al. 2012) with a GTR+I+G model, and 5,000,000 generations. Trees were sampled every 100 generations; four Markov chains were conducted over two runs, and the other parameters were established as default values. We excluded the first 25% of every tree, and the remaining trees were employed to produce a 50% majority consensus tree to estimate posterior probabilities. A bootstrap analysis with 1000 replicates was conducted to estimate the confidence of the tree nodes, and a bootstrap percentage (BP) of 50% or Bayesian posterior probability (BPP) of 0.9 was considered supportive across all constructed trees in this study.

## ﻿Results

### ﻿Yeast isolation

From the six samples of pulque aguamiel, forty-one phenotypically distinct colonies were chosen, and all were confirmed to be yeasts via microscopic analysis and uniform yeast colonies grew when plated on YM plates. The D1/D2 domain of the LSU rRNA gene were sequenced to characterize each yeast isolate. The nearest relative of each strain was recognized according to a BLAST search against the GenBank database using the BLASTn tool. The strains were classified to the species level based on a threshold of > 99% sequence identity with the type strain of a described species within the ITS region or D1/D2 domain (Kurtzman and Robnett 1998; Fell et al. 2000; Vu et al. 2016). A total of 41 strains were identified, clustered into 12 species belonging to 11 genera, the list of the isolated strains is shown in Table 1. Among the species identification, two were previously found in Pulque species *Kluyveromycesmarxianus* and *Saccharomycesparadoxus*, ten species not previously detected in Pulque, including seven known species *Apiotrichummycotoxinivorans*, *Candidaboidinii*, *Debaryomycesnepalensis*, *Papiliotremalaurentii*, *Pichiamanshurica*, *Piskurozymataiwanensis* and *Rhodosporidiobolusruineniae*, and three novel species *Kazachstania* sp., *Pichia* sp., and *Starmerella* sp.

**Table 1.** Strains isolated from samples of the traditional Mexican alcoholic beverage Pulque.

Location	Sample	Strain (NYNU)	Species
I	1	16111	*Apiotrichummycotoxinivorans*
	2	16113	
II	4	161140	
I	1	16116	*Candidaboidinii*
	3	161115	
II	4	161139	
	5	161147	
I	1	16118	*Debaryomycesnepalensis*
	3	161127	
I	1	**16111^1^**T	***Kazachstania* sp.**
	2	161114	
	3	161129	
I	1	16114	*Kluyveromycesmarxianus*
	2	161112	
	3	161113	
II	4	161141	
	5	161145	
	6	161146	
I	2	161118	*Papiliotremalaurentii*
	3	161131	
II	5	161143	
	6	161149	
I	1	161110	*Pichiamanshurica*
	2	161116	
	3	161130	
II	4	161138	
I	2	**16111^9^**T	***Pichia* sp.**
	3	161117	
II	5	161142	
	6	161153	
II	4	161136	*Piskurozymataiwanensis*
	6	161150	
I	1	16112	*Rhodosporidiobolusruineniae*
	3	161125	
II	4	161135	*Saccharomycesparadoxus*
	5	161137	
	6	161158	
I	1	161162	
I	1	**1611^5^**T	***Starmerella* sp.**
	2	161124	
	3	161128	

Location I: Teotihuacan in the State of Mexico (19°41'N, 98°50'W); Location II: Mexico City in the State of Distrito Federal (19°28'N, 99°09'W). NYNU, Microbiology Laboratory, Nanyang Normal University, Nanyang, Henan, China; T, type strain.

### ﻿Phylogeny

Among the yeast strains isolated, ten were unidentified as any species because of their significant difference from any described yeast species in the D1/D2 domain sequence. The ITS sequences of these strains were then determined to validate their novelty and phylogenetic positions.

**Figure 1.**
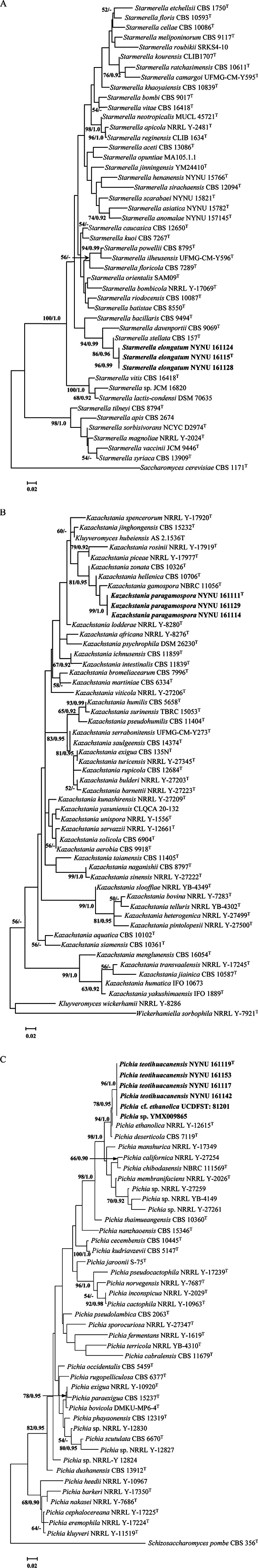
Phylogenetic trees derived from maximum-likelihood analysis based on the concatenated sequences of the ITS and D1/D2 domain of LSU rRNA, illustrating the positions of the novel yeast species in the genera *Starmerella* (**A**), *Kazachstania* (**B**) and *Pichia* (**C**). The tree backbones were developed using MEGA 7.0. The bootstrap support values (BS) ≥ 50% from ML analysis and Bayesian posterior probabilities (BPP) ≥ 0.90 are depicted on the branches, and a dash (“-”) indicates a value < 0.90 (BPP). Newly described species are indicated in bold. Scale bar, 2% sequence difference. T, type strains.

Three strains, NYNU 161124, NYNU 161128, and NYNU 16115^T^, isolated from two samples in Teotihuacan in the State of Mexico (Table 1), possessed identical sequences in the ITS regions and D1/D2 domain. They clustered with *Starmerellastellata* CBS 157^T^ and *S.davenportii* CBS 9069^T^ in the *Starmerella* clade (Fig. 1A). The D1/D2 sequences of the three strains differed from *S.stellata* CBS 157^T^ by ~1.0% sequence divergence (4 nucleotide (nt) substitutions and 1 gap out of 482 bp), differed from *S.davenportii* CBS 9069^T^ by ~7.0% sequence divergence (31 nt substitutions and 1 gap). In the ITS regions, the three strains displayed ~5.0% sequence divergence (16 nt substitutions and 5 gaps out of 433 bp) from *S.stellata*, exhibited ~10.0% mismatches (34 nt substitutions and 11 gaps) with *S.davenportii*. Thus, the three strains represent a novel *Starmerella* species, for which the name *S.elongatum* sp. nov. is proposed.

Three strains, NYNU 161111^T^, NYNU 161114, and NYNU 161129, with identical ITS and D1/D2 sequences, were located in the *Kazachstania* clade and closely associated with *K.gamospora* NBRC 11056^T^, *K.zonata* CBS 10326^T^, and *K.hellenica* CBS 10706^T^ in the tree derived from the combined ITS and D1/D2 sequences (Fig. 1B). The D1/D2 sequences of the three strains differed by 4 nt substitutions and 2 gaps (~1.0%) from *K.gamospora*, by 10 nt substitutions and 1 gap (~2.0%) from *K.hellenica*, and also by 10 nt substitutions and 1 gap (~2.0%) from *K.zonata* out of 573 bp. The ITS sequences of the three strains demonstrated a ~7.0–16% sequence divergence (29–64 nt substitutions and 19–55 gaps out of 712 bp) from *K.gamospora* NBRC 11056^T^, *K.hellenica* CBS 10706^T^, and *K.zonata* CBS 10326^T^, respectively. The findings suggest that the three strains represent a novel species in the *Kazachstania* genus, for which the name *K.paragamospora* sp. nov. is proposed.

Four strains, NYNU 161119, NYNU 161117, NYNU 161142, NYNU 161153, isolated from four samples (Table 1). The four strains and two unpublished strains labeled as Pichiacf.ethanolica UCDFST: 81-201 and *Pichia* sp. YMX009865 contained identical sequences in the D1/D2 domains, and the latter two strains had one nucleotide substitution with the four trains in the ITS regions, which indicated that they are conspeciﬁc. The closest relative of the six strains is *P.ethanolica* NRRL Y-12615^T^ (Fig. 1C), but differed from the strain of the latter by 5 nt substitutions out of 559 bp (~1%) in the D1/D2 domains and 19 nt substitutions and 7 gaps out of 398 bp (~7.0%) in the ITS regions. They also exhibited a close relationship with *P.deserticola* CBS 7119^T^ in the tree (Fig. 1C), they differed from the strain of the latter by 7 nt substitutions and 3 gaps (~2.0%) in the D1/D2 domains but had 20 nt substitutions and 10 gaps (~8.0%) mismatches in the ITS regions. Therefore, a novel species, *Pichiateotihuacanensis* sp. nov., is proposed to accommodate the six strains.

### ﻿Taxonomy

#### ﻿ Starmerella elongatum

Taxon classificationFungiSaccharomycetalesDebaryomycetaceae

C.Y. Chai & F.L. Hui sp. nov.

AE363AD4-F5AF-5055-96CB-E5503A456F38

841210

Fig. 2A, B

##### Etymology.

The specific epithet *elongatum* refers to the elongate vegetative cells of this yeast.

##### Type.

Mexico • State of Mexico, Teotihuacan, in the traditional Mexican alcoholic beverage Pulque, autumn of 2015, F.L. Hui, NYNU 16115 (holotype CICC 33262^T^ preserved in a metabolically inactive state, culture ex-type CBS 15224).

##### Description.

**Figure 2.**
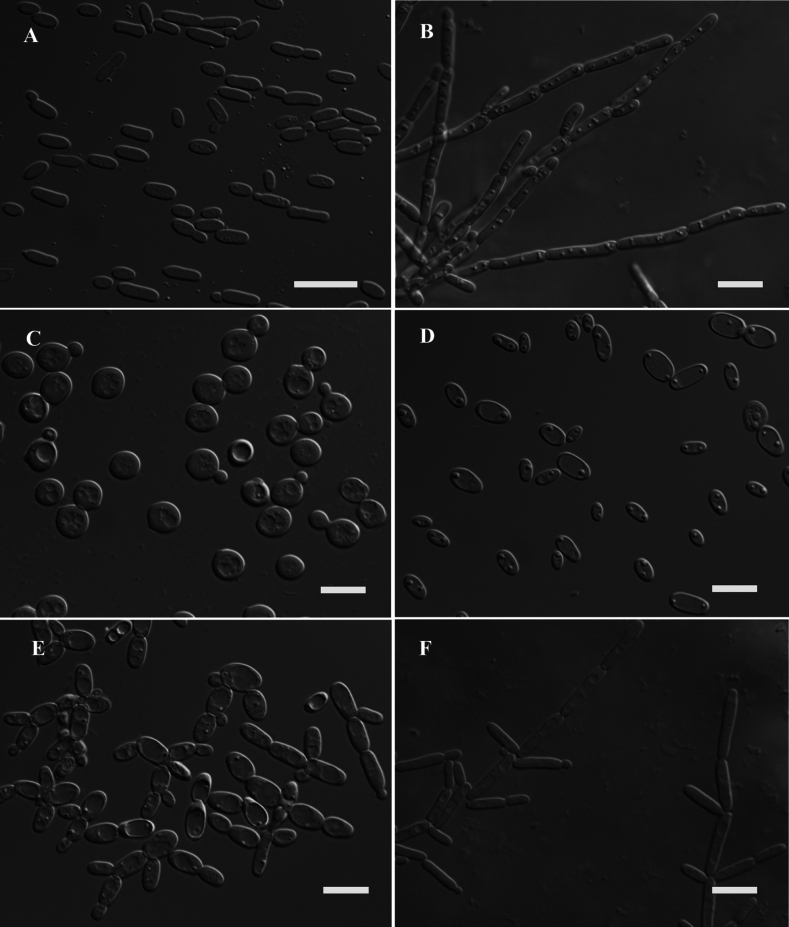
Morphological characteristics of *Starmerellaelongatum* sp. nov. NYNU 161115^T^**A** budding yeast cells, three days, YM broth, 25 °C **B** pseudohyphae grown on CM agar after 14 d at 25 °C. *Kazachstaniaparagamospora* sp. nov. NYNU 161111^T^**C** budding yeast cells in YM broth after 3 d, 25 °C. *Pichiateotihuacanensis* sp. nov. NYNU 161119^T^**D** budding yeast cells, three days, YM broth, 25 °C **E** simple pseudohyphae and **F** pseudohyphae grown on CM agar over 14 days at 25 °C. Scale bars: 10 μm.

After 3 days growth in YM broth at 25 °C, the cells are mostly ellipsoidal to elongate (2.2–3.4 × 3.9–7.3 μm) and occurred singly or in pairs. Budding is multilateral (Fig. 2A). Sediment is formed after one month, but no pellicle is observed. After 6 days culture on YM agar at 25 °C, colonies are white-cream in color, butyrous, smooth, and convex with complete margins. After 2 weeks in Dalmau plate culture on corn meal agar at 25 °C, pseudohyphae are present, but no true hyphae are observed (Fig. 2B). Asci or signs of conjugation are not seen on sporulation media. Glucose, sucrose, raffinose, and inulin (weakly) are fermented, while trehalose, galactose, maltose, melibiose, lactose, cellobiose, melezitose, methyl *α*-D-glucoside, soluble starch, or xylose are not. Glucose, sucrose (weakly), raffinose (weakly), inulin (weakly) and soluble starch (weakly) are assimilated. No growth occurred using as sole carbon source of melibiose, galactose, lactose, L-sorbose, L-rhamnose, L-arabinose, D-arabinose, D-ribose, methanol, ethanol, erythritol, galactitol, *myo*-inositol, DL-lactate, D-gluconate, xylitol, D-glucuronate, D-glucono-1,5-lactone, L-arabinitol, trehalose, maltose, melezitose, methyl *α*-D-glucoside, cellobiose, salicin, D-xylose, glycerol, ribitol, mannitol, glucitol, succinate, citrate, D-glucosamine, 2-keto-D-gluconate, arbutin, or 5-keto-D-gluconate. With respect to the assimilation of nitrogen compounds, L-lysine (weakly), cadaverine (weakly) and D-tryptophan (weakly) were assimilated, whereas nitrate, nitrite, ethylamine, creatine, creatinine, glucosamine and imidazole were not assimilated. Growth is observed at 30 °C but not at 35 °C. Growth is observed in the presence of 10% NaCl plus 5% glucose, 1% acetic acid, and vitamin-free medium, but not in the presence of 0.1% cycloheximide or 0.01% cycloheximide. Starch-like compounds are not produced. Urease activity and diazonium blue B reactions are also negative.

##### Additional strains examined.

Mexico • Teotihuacan in the State of Mexico (19°41'N, 98°50'W), in the traditional Mexican alcoholic beverage Pulque samples, autumn of 2015, F.L. Hui, NYNU 161124, and NYNU 161128.

##### GenBank accession numbers.

Holotype CICC 33262^T^ (ITS: MF136069, D1/D2: MF136061); additional strains NYNU 161124 (ITS: OM669948, D1/D2: OM669942) and NYNU 161128 (ITS: OM669943, D1/D2: OM670017).

##### Note.

*Starmerellaelongatum* sp. nov. can be physiologically differentiated from their nearest phylogenetic neighbor, *S.stellata* (Krumbholz 1931), on the basis of their ability to grow in the presence of 10% NaCl plus 5% glucose, 1% acetic acid, and vitamin-free medium, and their ability to assimilate inulin, soluble starch, and cadaverine (Table 2).

**Table 2.** Physiological characteristics differentiating three novel species from closely related species.

	Fermentation				Assimilation reactions and other characteristics																				
Species	D-Glucose	D-Galactose	Raffinose	Inulin	D-Glucose	Inulin	L-Sorbose	Soluble starch	Cadaverine	D-Galactose	L-Arabinose	Trehalose	L-Arabinitol	DL-Lactate	Succinate	Ethanol	Methanol	D-Glucosamine	Citrate	Ethylamine	Glucosamine	10%Nacl/5%glucose	Acetic acid 1%	Growth in vitamin-free edium	Growth at 30 °C
*S.stellata* ^[a]^	+	-	+	+	+	w	v	-	-	-	-	-	-	-	-	-	-	-	-	-	-	-	-	-	+
*S.elongatum* sp. nov.	+	-	+	w	+	-	-	w	w	-	-	-	-	-	-	-	-	-	-	-	-	w	+	+	+
*K.gamospora* ^[b]^	+	-	+	-	+	+	-	-	-	-	-	+	-	+	-	+	w	-	-	+	-	+	-	-	+
*K.zonata* ^[c]^	+	-	+	-	+	+	-	-	+	-	-	-	+	+	-	+	+	-	-	+	-	-	-	+	+
*K.hellenica* ^[d]^	+	+	-	+	+	+	-	+	+	+	+	d	+	-	+	-	w	-	-	-	-	-	-	-	-
*K.paragamospora* sp. nov.	+	-	+	+	+	+	-	w	w	-	-	+	w	-	w	-	w	-	-	-	-	-	-	+	+
*P.ethanolica* ^[e]^	s	-	-	-	-	-	-	-	+	-	-	-	-	w	w	+	-	-	-	+	-	-	-	+	+
*P.deserticola* ^[f]^	-	-	-	-	-	-	-	-	-	-	-	-	-	+	+	+	-	-	-	-	-	-	+	-	+
*P.teotihuacanensis* sp. nov.	-	-	-	-	-	w	-	w	-	-	-	-	-	w	w	-	w	+	w	-	w	w	+	+	+

^[a]^ Data from Foschino et al. (2004); ^[b,c]^ Data from Imanishi et al. (2007); ^[d]^ Data from Nisiotou and Nychas (2008); ^[e]^ Data from Rybářová et al. (1980); ^[f]^ Data from Kurtzman et al. (2008). +, Positive; −, negative; w, weakly positive; s, slow positive; d, delayed positive; v, variable.

#### ﻿ Kazachstania paragamospora

Taxon classificationFungiSaccharomycetalesDebaryomycetaceae

C.Y. Chai & F.L. Hui sp. nov.

8BE1280B-1B30-55C0-B659-209F85F37941

841211

Fig. 2C

##### Etymology.

the specific epithet *paragamospora*, like *gamospora*, referring to its phylogenetic closeness to *Kazachstaniagamospora*.

##### Type.

Mexico • State of Mexico, Teotihuacan, in the traditional Mexican alcoholic beverage Pulque, autumn of 2015, F.L. Hui, NYNU 161111 (holotype CICC 33274^T^ preserved in a metabolically inactive state, culture ex-type CBS 15233).

##### Description.

After 3 days culture in YM broth at 25 °C, the cells are spherical or ovoid (3–6.2 × 3.3–7.5 μm) and occurred singly or in pairs (Fig. 2C). Budding is multilateral. Sediment is formed after one month, but no pellicle is observed. After 3 days cultured on YM agar at 25 °C, the streak culture is butyrous, white, raised with a smooth surface, and has a complete margin. Asci or signs of conjugation are not observed on sporulation media. Glucose, sucrose, raffinose, and inulin are fermented, while trehalose, galactose, maltose, melibiose, lactose, cellobiose, melezitose, methyl *α*-D-glucoside, soluble starch, or xylose are not. Glucose, sucrose (weakly), raffinose (weakly), inulin, glycerol (weakly), L-arabinitol (weakly), 5-keto-D-gluconate (weakly), D-gluconate, D-glucosamine (weakly), succinate (weakly), methanol (weakly) and soluble starch (weakly) are assimilated. No growth occurred in the presence of melibiose, galactose, lactose, L-sorbose, L-rhamnose, L-arabinose, D-arabinose, D-ribose, ethanol, erythritol, galactitol, *myo*-inositol, DL-lactate, xylitol, D-glucuronate, D-glucono-1,5-lactone, trehalose, maltose, melezitose, methyl α-D-glucoside, cellobiose, salicin, D-xylose, ribitol, mannitol, glucitol, citrate, 2-keto-D-gluconate, or arbutin. With respect to the assimilation of nitrogen compounds, cadaverine (weakly) and D-tryptophan are assimilated, whereas L-lysine, nitrate, nitrite, ethylamine, creatine, creatinine, glucosamine, and imidazole are not. Growth is observed at 30 °C but not at 35 °C. Growth in the presence of vitamin-free medium is positive, but growth in the presence of 10% NaCl plus 5% glucose, 1% acetic acid, 0.1% cycloheximide, and 0.01% cycloheximide are negative. Starch-like compounds are not produced. Urease activity and diazonium blue B reactions are also negative.

##### Additional strains examined.

Mexico • Teotihuacan in the State of Mexico (19°41'N, 98°50'W), in the traditional Mexican alcoholic beverage Pulque samples, autumn of 2015, F.L. Hui, NYNU 161114 and NYNU 161129.

##### GenBank accession numbers.

Holotype CICC 33274^T^ (ITS: MF136070, D1/D2: MF136062); additional strains NYNU 161114 (ITS: OM669944, D1/D2: OM669945) and NYNU 161129 (ITS: OM669946, D1/D2: OM669947).

##### Note.

*Kazachstaniaparagamospora* sp. nov. can be differed from the related three species *K.gamospora*, *K.zonata* and *K.hellenica* by its inability to assimilate trehalose (Table 2). Unlike *K.hellenica*, the novel species was able to ferment raffinose and grow at 30 °C but was not able to ferment galactose. Likewise, the novel species did not assimilate maltose and D-galactose. The novel species differed from *K.zonata* by its inability to assimilate ethylamine, ethanol, and DL-lactate. It differed from *K.gamospora* in its ability to assimilate L-arabinitol and cadaverine (weakly), and inability to grow in the presence of 10% NaCl plus 5% glucose. In all cases, identification by sequencing was the best approach.

#### ﻿ Pichia teotihuacanensis

Taxon classificationFungiSaccharomycetalesDebaryomycetaceae

C.Y. Chai & F.L. Hui sp. nov.

2CFC2200-F03C-5748-A9D0-767C65D9886C

841212

Fig. 2D, E, F

##### Etymology.

The specific epithet teotihuacan of or belonging to the State of Mexico, the geographical origin of the type strain of the isolated species.

##### Type.

Mexico • State of Mexico, Teotihuacan, in the traditional Mexican alcoholic beverage Pulque sample, autumn of 2015, F.L. Hui, NYNU 161119 (holotype CICC 33275^T^ preserved in a metabolically inactive state, culture ex-type CBS 15277).

##### Description.

After 3 days culture in YM broth at 25 °C, the cells are ovoid (2.5–7.0 × 3.7–9.5 µm) and occurred singly or in pairs. Budding is multilateral (Fig. 2D). After 6 days cultured on YM agar at 25 °C, colonies are cream colored, butyrous and rough, with filamentous margins. After 2 weeks in Dalmau plate culture on corn meal agar at 25 °C, pseudohyphae formed but true hyphae did not (Fig. 2E, F). Ascospores are not observed on YM, 5% malt extract, cornmeal, and YCBAS agar media in pure and mixed cultures at 17 °C and 25 °C for up to 4 weeks. Sugar fermentation is absent. Glucose, D-glucosamine, inulin (weakly), soluble starch (weakly), glycerol (weakly), DL-lactate (weakly), succinate (weakly), citrate (weakly), and methanol (weakly) are assimilated. No growth occurred using as sole carbon of melibiose, sucrose, raffinose, L-arabinitol, 5-keto-D-gluconate, D-gluconate, galactose, lactose, L-sorbose, L-rhamnose, L-arabinose, D-arabinose, D-ribose, ethanol, erythritol, galactitol, *myo*-inositol, xylitol, D-glucuronate, D-glucono-1,5-lactone, trehalose, maltose, melezitose, methyl *α*-D-glucoside, cellobiose, salicin, D-xylose, ribitol, mannitol, glucitol, 2-keto-D-gluconate, or arbutin. With respect to the assimilation of nitrogen compounds, L-lysine, glucosamine (weakly), and D-tryptophan were assimilated, whereas nitrate, nitrite, ethylamine, creatine, adaverine, creatinine, and imidazole were not. Growth is observed at 37 °C but not at 40 °C. Growth in the presence of vitamin-free medium, 10% NaCl plus 5% glucose, and 1% acetic acid are positive, while growth in the presence of 0.1% cycloheximide or 0.01% cycloheximide is negative. Starch-like compounds are not produced. Urease activity and diazonium blue B reactions are also negative.

##### Additional strains examined.

Mexico • Teotihuacan in the State of Mexico (19°41'N, 98°50'W) and Mexico City in the State of Distrito Federal (19°28'N, 99°09'W), in the traditional Mexican alcoholic beverage Pulque samples, autumn of 2015, F.L. Hui, NYNU 161117, NYNU 161142 and NYNU 161153.

##### GenBank accession numbers.

Holotype CICC 33275^T^ (ITS: MF136068, D1/D2: MF136064); additional strains NYNU 161117 (ITS: OM670016, D1/D2: OM670012), NYNU 161142 (ITS: OM670015, D1/D2: OM670013) and NYNU 161153 (ITS: OM670079, D1/D2: OM670014).

##### Note.

*Pichiateotihuacanensis* sp. nov. can be physiologically differentiated from *P.ethanolica* in terms of positive assimilation of inulin, D-glucosamine, soluble starch, citrate, methanol, and glucosamine, and an inability to assimilate ethanol, ethylamine, and cadaverine. *P.teotihuacanensis* differed from *P.deserticola*, in terms of their ability to assimilate D-glucosamine, inulin, and soluble starch, and grow in vitamin-free medium (Table 2).

## ﻿Discussion

As previously described, Pulque is a critically important, traditional, non-distilled alcoholic beverage produced in the central states of Mexico. Pulque is the focus of research across numerous laboratories, not only due to its nutritional properties but also to the complex microbial diversity responsible for fermentation, a complex procedure that has proven recalcitrant to industrialization. While various microbial studies regarding Pulque have been performed over the last 100 years, the complex yeast found within the beverage has remained uncharacterized. Recently, several yeast species have been identified in Pulque, such as *Candidavalida*, *C.zemplinina*, *C.stellata*, *C.parapsilosis*, *Clavisporalusitaniae*, *Debaryomicescarsonii*, *Geotrichumcandidum*, *Hanseniasporauvarum*, *Kazachstanialactis*, *Kluyveromycesmarxianus*, *Pichiaguilliermondii*, *P.membranifaciens*, *Rhodotorulamucilaginosa*, Saccharomycescerevisiaesubsp.chevalieri, S.cerevisiaesubsp.capensis, *S.bayanus*, *S.paradoxus*, *Torulasporadelbrueckii* and so on (Ruiz Oronoz 1953; Lappe and Ulloa 1993; Estrada-Godina et al. 2001; Lappe-Oliveras et al. 2008; Escalante et al. 2016; Rocha-Arriaga et al. 2020). However, a large number of undescribed yeast taxa in Pulque remain.

In this study, we collected six Pulque samples from two “*pulquerías*” (Pulque stores) in two different regions in Mexico. From these samples, we extracted forty-one yeast strains, clustered into 12 yeast species belonging to 11 genera. Of the total isolates, there were nine representatives of previously known species, like *Apiotrichummycotoxinivorans*, *Candidaboidinii*, *Debaryomycesnepalensis*, *Kluyveromycesmarxianus*, *Papiliotremalaurentii*, *Pichiamanshurica*, *Piskurozymataiwanensis*, *Rhodosporidiobolusruineniae* and *Saccharomycesparadoxus*. In addition to known species, we recovered ten strains belonging to 3 yeast species distinct from any previously described, including *Kazachstania* sp., *Pichia* sp., and *Starmerella* sp. Three more comprehensive separate phylogenetic placements of the genera *Kazachstania*, *Pichia*, and *Starmerella* according to the combined ITS and LSU rDNA sequences are provided. Each phylogenetic tree includes almost all GenBank representatives and newly generated sequences, potentially serving as the references for the three fields. Thereafter, we described these novel species as *Starmerellaelongatum* sp. nov., *Kazachstaniaparagamospora* sp. nov., and *Pichiateotihuacanensis* sp. nov. based on molecular phylogenetic and morphological evidence.

According to the details previously reported, yeast plays an important role in the fermentation of Pulque, producing ethanol, amino acids, vitamins, and volatile compounds. Among the total isolates, two known species, *Klu.marxianus* and *S.paradoxus* were found to occur in Pulque, *Klu.marxianus* can excrete enzymes and be found within Pulque, can ferment at high temperatures (45 °C), use complex sugars such as inulin and hemicellulose, and has been used to generate bioethanol with a broader substrate range and higher temperature tolerance (Sofia et al. 2015). Additionally, *Saccharomycesparadoxus*, a native microorganism in the Pulque, was used successfully for endoinulinase synthesis for agave fructooligosaccharide (FOS) production (Sandoval-González et al. 2018). The remaining seven known species (*A.mycotoxinivorans*, *C.boidinii*, *D.nepalensis*, *Pap.laurentii*, *P.manshurica*, *Pis.taiwanensis* and *Rho.ruineniae*), alongside three novel species (*K.paragamospora*, *P.teotihuacanensis* and *S.elongatum*) had not been reported in Pulque previously, the biochemical role of the ten newly isolated species from the Pulque fermentation process remains unknown. These species may be responsible for Pulque fermentation through specific biological characteristics. For instance, *Candidaboidinii* can excrete alcohol oxidase (Ciani et al. 2000). *Papiliotremalaurentii* produces extracellular *β*-glucosidase, amylase, protease, lipase, phytase, and xylanase (Vieira et al. 2020). We isolated two *Pichia* yeast species, including *P.manshurica* and the novel species *P.teotihuacanensis* sp. nov. Yeasts of the *Pichia* genus are associated with fermentation processes, and are considered to be wine-related yeasts, producing alcohol, organic acids, and esters (Pang et al. 2018; Tan et al. 2019). *P.manshurica* is a common yeast identified in fermented animal feed and foods, including fermented maguey juice, a traditional beverage in Burkina Faso, wines, Ishizuchi-kurocha, and silages (Toyotome et al. 2019), with important roles in fermentation and decomposing plant materials. *P.ethanolica*, the closest relative of *P.teotihuacanensis* sp. nov., is an endogenous yeast of the *Agave* plant. This study increases the number of described *Pichia* species identified in *pulque*. *Apiotrichummycotoxinivorans* exhibits cellulase and amylase production capabilities, which have been isolated in the Iguaçu National Park and used for the production of xylanolytic enzymes to be used for ethanol synthesis (Carvalho et al. 2021). *Kazachstania* species have been isolated from fermented fermented foods and silage (Ke et al. 2019), i.e., *K.solicola*—vinegar and solvents; *K.hellenica*—spirits and cheeses; and *K.aerobia*—imparting a fruity and ﬂoral flavor (Illse et al. 2017). *K.gamospora*, the closest relative of the novel species *K.paragamospora* sp. nov., is associated with producing alcohols, ketones, certain aldehydes, and acetate esters. Some of the *Starmerella* species are particularly associated with sweet, botrytized, wine fermentations; for example, *S.bacillaris*, *S.cerevisiae and S.uvarum* are frequently isolated from wine-related sources (Magyar and Tóth 2011). *S.stellata*, the closest relative of the novel species *S.elongatum* sp. nov., is often found in wine fermentations and may be used as a yeast starter for beverage production. However, the biochemical role of the novel species in the *pulque* fermentation remains unclear. *Debaryomycesnepalensis*, an osmotolerant yeast isolated from rotten apples, uses both hexoses and pentoses generates industrially relevant metabolites, such as ethanol, xylitol, and arabitol (Himabindu et al. 2013). There are limited reports about *P.taiwanensis*, notably only documented by Satianpakiranakorn et al. (2020) as an antagonistic yeast strain. Its documentation in so few reports suggests that it occurs at low frequency. *R.ruineniae* was reported by Ramírez-Castrillón et al. (2019) and Maciel et al. (2013), which was isolated from coconut water and reconstituted juices from Belo Horizonte, Brazil. Despite the limited number of yeast strains identified in our survey, they may still be responsible for Pulque fermentation through their unique biological characteristics.

This study reports on the isolation of yeast species not previously identified in Pulque, extending our understanding of the genetic diversity of its associated yeast communities. To date, alongside the three newly isolated species in this study, more and more yeast species have been isolated from Pulque. Although the yeast taxonomy of Pulque has been a focus of research in the past, the native microbial community is likely related to the traditional non-aseptic conditions used during the collection, transportation, and fermentation; there are still some species that require discovery in Pulque in the future studies.

## Supplementary Material

XML Treatment for Starmerella elongatum

XML Treatment for Kazachstania paragamospora

XML Treatment for Pichia teotihuacanensis

## ﻿Additional information

### Author ORCIDs

Chunyue Chai https://orcid.org/0000-0003-0284-5560

Fengli Hui https://orcid.org/0000-0001-7928-3055

### Data availability

All of the data that support the findings of this study are available in the main text or Supplementary Information.

## Supplementary materials

Supplementary material 1 DNA sequences used in the molecular phylogenetic analysis of *Starmerella* genus Data type docx https://binary.pensoft.net/file/1150642This dataset is made available under the Open Database License (http://opendatacommons.org/licenses/odbl/1.0/). The Open Database License (ODbL) is a license agreement intended to allow users to freely share, modify, and use this Dataset while maintaining this same freedom for others, provided that the original source and author(s) are credited.Chunyue Chai, Dan Lu, Jinli Liu, Eentao Wang, Xuemei Han, Fengli Hui

Supplementary material 2 DNA sequences used in the molecular phylogenetic analysis of *Kazachstania* genus Data type docx https://binary.pensoft.net/file/1150643This dataset is made available under the Open Database License (http://opendatacommons.org/licenses/odbl/1.0/). The Open Database License (ODbL) is a license agreement intended to allow users to freely share, modify, and use this Dataset while maintaining this same freedom for others, provided that the original source and author(s) are credited.Chunyue Chai, Dan Lu, Jinli Liu, Eentao Wang, Xuemei Han, Fengli Hui

Supplementary material 3 DNA sequences used in the molecular phylogenetic analysis of *Pichia* genus Data type docx https://binary.pensoft.net/file/1150644This dataset is made available under the Open Database License (http://opendatacommons.org/licenses/odbl/1.0/). The Open Database License (ODbL) is a license agreement intended to allow users to freely share, modify, and use this Dataset while maintaining this same freedom for others, provided that the original source and author(s) are credited.Chunyue Chai, Dan Lu, Jinli Liu, Eentao Wang, Xuemei Han, Fengli Hui
