# Supplementary material for: ﻿Three novel Ascomycota (Saccharomycetes, Saccharomycetales) yeast species derived from the traditional Mexican alcoholic beverage Pulque
Source: MycoKeys. 2024 Oct 9;109:187–206. doi: 10.3897/mycokeys.109.123870 (PMC11484639; doi:10.3897/mycokeys.109.123870)
Supplement: Supplementary material 1 — DNA sequences used in the molecular phylogenetic analysis of Starmerella genus [file mycokeys-109-187-s001.docx]

**Supplementary Table** S1 DNA sequences used in the molecular phylogenetic analysis of *Starmerella* genus. Entries in bold were newly generated for this study.

| Taxa name | Strain-number | GenBank accession numbers | |
| --- | --- | --- | --- |
|  |  | ITS | LSU D1/D2 |
| *Starmerella etchellsii* | CBS 1750^T^ | KY102077 | KY106442 |
| *S. floris* | CBS 10593^T^ | KY102087 | AF313353 |
| *S. cellae* | CBS 10086^T^ | KY102026 | KY106391 |
| *S. meliponinorum* | CBS 9117^T^ | KY105547 | AF313354 |
| *S. roubikii* | CBS 15148 | MF668211 | LC646114 |
| *S. kourensis* | CLIB 2707 | LN909469 | LN909483 |
| *S. ratchasimensis* | CBS 10611^T^ | KY102359 | AY228492 |
| *S. camargoi* | UFMG-CM-Y595 | N/A | KR232373 |
| *S. khaoyaiensis* | CBS 10839^T^ | KY102169 | DQ400367 |
| *S. bombi* | CBS 9017^T^ | AB696986 | AF406929 |
| *S. vitae* | UWOPS 00-107.2^T^ | NR 173262 | KX418642 |
| *S. neotropicalis* | MUCL 45721^T^ | FM209493 | FM209492 |
| *S. apicola* | NRRL Y-2481^T^ | NR 130681 | NG 075433 |
| *S. reginensis* | CLIB 1634^T^ | LN870338 | LN870357 |
| *S. aceti* | CBS 13086^T^ | KF271437 | KF247224 |
| *S. opuntiae* | MA105.1.1 | KJ410157 | KJ410156 |
| *S. jinningensis* | YM24410^T^ | KY105546 | KY109784 |
| *S. henanensis* | NYNU 15766^T^ | KU128716 | KU128730 |
| *S. sirachaensis* | CBS 12094^T^ | KY102396 | AB617909 |
| *S. scarabaei* | NYNU 15821^T^ | KU128718 | KU 128729 |
| *S. asiatica* | NYNU 15782^T^ | KU128719 | KU128728 |
| *S. anomalae* | NYNU 157145^T^ | KU128717 | KU128732 |
| *S. caucasica* | CBS 12650^T^ | JX112044 | JX112043 |
| *S. kuoi* | CBS 7267^T^ | NR 164377 | HM104577 |
| *S. powellii* | CBS 8795^T^ | KY102339 | AF251554 |
| *S.* *ilheusensis* | UFMG-CM-Y596^T^ | KR232375 | KR232374 |
| *S. floricola* | CBS 7289^T^ | KY102086 | KY106448 |
| *S. orientalis* | SAM09^T^ | KM269181 | ON454127 |
| *S. bombicola* | NRRL Y-17069^T^ | NR 121483 | U45705.1 |
| *S. riodocensis* | CBS 10087^T^ | KY102365 | KJ630496.1 |
| *S. batistae* | CBS 8550^T^ | KY101955 | AF072843 |
| *S. bacillaris* | CBS 9494^T^ | OP663284 | KY106894 |
| *S. davenportii* | CBS 9069^T^ | KY102043 | AJ310447 |
| *S. stellata* | CBS 157^T^ | ON318880 | U45730 |
| ***S. elongatum*** | **NYNU 16115^T^** | **MF136069** | **MF136061** |
| ***S. elongatum*** | **NYNU 161124** | **OM669948** | **OM669942** |
| ***S. elongatum*** | **NYNU 161128** | **OM669943** | **OM670017** |
| *S. vitis* | CBS 16418^T^ | MN317383 | KC992848 |
| *Starmerella* sp. | JCM 16820 | AB565762 | AB565749 |
| *S. lactis-condensi* | CBS 2633 | OL679484 | U45724 |
| *S. tilneyi* | CBS 8794^T^ | KJ630493 | AF251553 |
| *S. apis* | CBS 2674^T^ | OW987779 | NG 060800 |
| *S. sorbisivorans* | CBS 3080 | MT845221 | AJ277846 |
| *S. magnoliae* | NRRL Y-2024^T^ | KJ707049 | NG 060814 |
| *S. vaccinii* | JCM 9446^T^ | KY611848 | KJ630499 |
| *S. syriaca* | CBS 13909^T^ | JX515987 | JX515986 |
| *Saccharomyces cerevisiae* | CBS 1171^T^ | KC881067 | KC881066 |

Notes: Type strains are marked with T; “N/A” means that sequences were not available.
