# Supplementary material for: ﻿Three novel Ascomycota (Saccharomycetes, Saccharomycetales) yeast species derived from the traditional Mexican alcoholic beverage Pulque
Source: MycoKeys. 2024 Oct 9;109:187–206. doi: 10.3897/mycokeys.109.123870 (PMC11484639; doi:10.3897/mycokeys.109.123870)
Supplement: Supplementary material 2 — DNA sequences used in the molecular phylogenetic analysis of Kazachstania genus [file mycokeys-109-187-s002.docx]

**Supplementary Table** S2 DNA sequences used in the molecular phylogenetic analysis of *Kazachstania* genus. Entries in bold were newly generated for this study.

| Taxa name | Strain-number | GenBank accession numbers | |
| --- | --- | --- | --- |
|  |  | ITS | LSU D1/D2 |
| *Kazachstania rosinii* | NRRL Y-17919^T^ | KY103665 | NG 055031 |
| *K. piceae* | NRRL Y-17977^T^ | KY103661 | KY107938 |
| *K. zonata* | CBS 10326^T^ | KY103696 | AB198187 |
| *K. hellenica* | CBS 10706^T^ | EU075202 | EF620861 |
| *K. gamospora* | CBS 10328^T^ | ON306659 | AB087388 |
| ***K.* *paragamospora*** | **NYNU 161111^T^** | **MF136070** | **MF136062** |
| ***K. paragamospora*** | **NYNU 161114** | **OM669944** | **OM669945** |
| ***K.* *paragamospora*** | **NYNU 161129** | **OM669946** | **OM669947** |
| *K. spencerorum* | NRRL Y-17920^T^ | KY103674 | AY048162 |
| *Kluyveromyces hubeiensis* | LYSM14^T^ | MW710012 | AY325967 |
| *K. jinghongensis* | CBS 15232^T^ | MG255727 | MG255708 |
| *K. lodderae* | NRRL Y-8280^T^ | KY103653 | AY048161 |
| *K. martiniae* | CBS 6334^T^ | OK052489 | AJ279063 |
| *K. africana* | NRRL Y-8276^T^ | KY103620 | NG 055030 |
| *K. psychrophila* | DSM 26230^T^ | JX656699 | JX564243 |
| *K. bromeliacearum* | CBS 7996^T^ | KY103627 | HQ412595 |
| *K. viticola* | NRRL Y-27206^T^ | ON969397 | AF398482 |
| *K. ichnusensis* | CBS 11859 | N/A | HQ616669 |
| *K. intestinalis* | CBS 11839^T^ | HQ260336 | HQ260337 |
| *K. solicola* | CBS 6904^T^ | KY103672 | AY007895 |
| *K. aerobia* | CBS 9918^T^ | AY582126 | AY582127 |
| *K. unispora* | NRRL Y-1556^T^ | OP984726 | AY048158 |
| *K. servazzii* | NRRL Y-12661^T^ | PP003906 | AY048157 |
| *K. yasuniensis* | CLQCA 20-132^T^ | MW895952 | HG934855 |
| *K. aquatica* | CBS 10102^T^ | KY103622 | NG 055046 |
| *K. siamensis* | CBS 10361^T^ | KY103669 | NG 042470 |
| *K. naganishii* | CBS 8797^T^ | KY103659 | KY107935 |
| *K. sinensis* | NRRL Y-27222^T^ | AY046167 | AF398484 |
| *K. taianensis* | CBS 11405^T^ | KY103675 | KY107953 |
| *K. slooffiae* | NRRL YB-4349^T^ | OP642535 | OR338212 |
| *K. bovina* | NRRL Y-7283^T^ | KY103626 | AY545571 |
| *K. telluris* | NRRL YB-4302^T^ | OP642538 | AY545569 |
| *K. heterogenica* | NRRL Y-27499^T^ | KY103646 | AY545578 |
| *K. pintolopesii* | NRRL Y-27500^T^ | KY103663 | AJ508579 |
| *K. kunashirensis* | NRRL Y-27209^T^ | OK051061 | AF398483 |
| *K. menglunensis* | CBS 16054^T^ | MK682820 | MK682819 |
| *K. transvaalensis* | NRRL Y-17245^T^ | KY103678 | U68549 |
| *K. jiainica* | CBS 10587^T^ | KY103648 | EF460568 |
| *K. humatica* | IFO 10673^T^ | AB097397 | AB040999 |
| *K. yakushimaensis* | IFO 1889^T^ | KY103692 | AB041000 |
| *K. pseudohumilis* | CBS 11404^T^ | KY102343 | FJ888526 |
| *K. humilis* | CBS 5658^T^ | OK247581 | KY106507 |
| *K. rupicola* | CBS 12684 | JQ861272 | N/A |
| *K. exigua* | CBS 135N^T^ | OR507600 | AJ508565 |
| *K. turicensis* | NRRL Y-27345^T^ | KY103679 | AF398485 |
| *K. bulderi* | NRRL Y-27203^T^ | OM996016 | KY107908 |
| *K. barnettii* | NRRL Y-27223^T^ | KY103624 | AY048164 |
| *K. serrabonitensis* | UFMG-CM-Y273^T^ | OK051519 | KF582608 |
| *K. surinensis* | TBRC 15053^T^ | LC628454 | LC626528 |
| *K. saulgeensis* | CBS 14374^T^ | NR 164574 | NG 055063 |
| *Kluyveromyces wickerhamii* | NRRL Y-8286^T^ | KY103845 | KY108112 |
| *Wickerhamiella sorbophila* | NRRL Y-7921^T^ | KY102412 | DQ438229 |

Notes: Type strains are marked with T; “N/A” means that sequences were not available.
