# Supplementary material for: ﻿Three novel Ascomycota (Saccharomycetes, Saccharomycetales) yeast species derived from the traditional Mexican alcoholic beverage Pulque
Source: MycoKeys. 2024 Oct 9;109:187–206. doi: 10.3897/mycokeys.109.123870 (PMC11484639; doi:10.3897/mycokeys.109.123870)
Supplement: Supplementary material 3 — DNA sequences used in the molecular phylogenetic analysis of Pichia genus [file mycokeys-109-187-s003.docx]

**Supplementary Table** S3 DNA sequences used in the molecular phylogenetic analysis of *Pichia* genus. Entries in bold were newly generated for this study.

| Taxa name | Strain-number | GenBank accession numbers | |
| --- | --- | --- | --- |
|  |  | ITS | LSU D1/D2 |
| *Pichia membranifaciens* | NRRL Y-2026^T^ | DQ104730 | EF550227 |
| *Pichia* sp. | NRRL YB-4149 | N/A | EF550224 |
| *Pichia* sp. | NRRL Y-27261 | N/A | EF550229 |
| *Pichia* sp. | NRRL Y-27259 | N/A | EF550228 |
| *P. californica* | NRRL Y-27254^T^ | KY102011 | EF550230 |
| *P. chibodasensis* | NBRC 111569^T^ | NR 153305 | LC126429 |
| *P. manshurica* | NRRL Y-17349^T^ | MW063524 | EF550223 |
| *P. deserticola* | NRRL Y-12918^T^ | AY790539 | EF550226 |
| *P. ethanolica* | NRRL Y-12615^T^ | AY790538 | EF550225 |
| ***P. teotihuacanensis*** | **NYNU 161119^T^** | **MF136068** | **MF136064** |
| ***P.* *teotihuacanensis*** | **NYNU 161117** | **OM670016** | **OM670012** |
| ***P.* *teotihuacanensis*** | **NYNU 161142** | **OM670015** | **OM670013** |
| ***P. teotihuacanensis*** | **NYNU 161153** | **OM670079** | **OM670014** |
| *P. thaimueangensis* | CBS 10360^T^ | KY102439 | AB264009 |
| *P. fermentans* | NRRL Y-1619^T^ | DQ665310 | EF550234 |
| *P. nanzhaoensis* | CBS 15346^T^ | MG255719 | MG255700 |
| *P. jaroonii* | S-75^T^ | N/A | AB436766 |
| *P. cecembensis* | CBS 10445^T^ | AM233511 | AM159112 |
| *P. kudriavzevii* | CBS 5147^T^ | KX015902 | U76347 |
| *P. pseudolambica* | CBS 2063^T^ | KY102346 | KY106706 |
| *P. sporocuriosa* | NRRL Y-27347^T^ | OR554041 | EF550232 |
| *P. dushanensis* | CBS 13912^T^ | KM272245 | KM272244 |
| *Pichia* sp. | NRRL-Y 12824 | N/A | AF017398 |
| *P. occidentalis* | CBS 5459^T^ | OR462339 | OP874757 |
| *P. exigua* | NRRL Y-10920^T^ | KY104525 | EF550237 |
| *P. paraexigua* | CBS 15237^T^ | MG255726 | MG255712 |
| *P. phayaonensis* | CBS 12319^T^ | KY102331 | AB557865 |
| *Pichia* sp. | NRRL Y-12830 | N/A | AF017399 |
| *P. rugopelliculosa* | CBS 6377^T^ | KY102367 | U71069 |
| *P. scutulata* | CBS 6670^T^ | KY104645 | EF550243 |
| *Pichia* sp. | NRRL Y-12827 | N/A | EF550245 |
| *P. terricola* | NRRL YB-4310 | LC790891 | EF550233 |
| *P. cabralensis* | CBS 11679^T^ | KY102010 | FJ755462 |
| *P. norvegensis* | NRRL Y-7687^T^ | MH396411 | EF550239 |
| *P. pseudocactophila* | NRRL Y-17239 | N/A | EF550242 |
| *P. inconspicua* | NRRL Y-2029^T^ | KY102148 | EF550240 |
| *P. cactophila* | NRRL Y-10963^T^ | OP764009 | EF550241 |
| *P. nakasei* | NRRL Y-7686^T^ | KY104633 | EF550248 |
| *P. kluyveri* | NRRL Y-11519^T^ | DQ104711 | EF550251 |
| *P. eremophila* | CBS 7272^T^ | KY104524 | EF550249 |
| *P. cephalocereana* | NRRL Y-17225^T^ | KY104518 | EF550250 |
| *P. barkeri* | NRRL Y-17350^T^ | KY104510 | EF550247 |
| *P. heedii* | NRRL Y-10967^T^ | KY104553 | EF550252 |
| *Schizosaccharomyces pombe* | CBS 356^T^ | N/A | AY048171 |

Notes: Type strains are marked with T; “N/A” means that sequences were not available.
